# Thrombolytic Effects of the Snake Venom Disintegrin Saxatilin Determined by Novel Assessment Methods: A FeCl_3_-Induced Thrombosis Model in Mice

**DOI:** 10.1371/journal.pone.0081165

**Published:** 2013-11-18

**Authors:** Il Kwon, Sung-Yu Hong, Young Dae Kim, Hyo Suk Nam, Sungsoo Kang, Seung-Hee Yang, Ji Hoe Heo

**Affiliations:** 1 Department of Neurology, Yonsei University College of Medicine, Seoul, Korea; 2 Severance Integrative Research Institute for Cerebral and Cardiovascular Diseases, Yonsei University College of Medicine, Seoul, Korea; 3 Severance Biomedical Science Institute, Yonsei University College of Medicine, Seoul, Korea; 4 Cardiovascular Product Evaluation Center, Yonsei University College of Medicine, Seoul, Korea; Institute of Neurology (Edinger-Institute), Germany

## Abstract

Saxatilin, a novel disintegrin purified and cloned from the venom of the Korean snake *Gloydius saxatilis*, strongly inhibits activation and aggregation of platelets. Glycoprotein (GP) IIb/IIIa receptor antagonists can resolve thrombus, so saxatilin might also have thrombolytic effects. We investigated the thrombolytic effects of saxatilin in mice using a ferric chloride-induced carotid arterial thrombosis model. Thrombotic occlusion and thrombus resolution were evaluated quantitatively by measuring blood flow in the carotid artery with an ultrasonic flow meter and calculating the degree of flow restoration on a minute-by-minute basis; results were confirmed by histological examination. Saxatilin dissolved thrombi in a dose-dependent manner. Saxatilin at 5 mg/kg restored blood flow to baseline levels. As saxatilin dose increased, time to recanalization decreased. A bolus injection of 10% of a complete dose with continuous infusion of the remaining dose for 60 minutes resulted in effective recanalization without reocclusion. The thrombolytic effect of saxatilin was also demonstrated *in vitro* using platelet aggregometry by administering saxatilin in preformed thrombi. Bleeding complications were observed in 2 of 71 mice that received saxatilin. Fibrin/fibrinogen zymography and platelet aggregometry studies indicated that saxatilin does not have fibrinolytic activity, but exerted its action on platelets. Integrin-binding assays showed that saxatilin inhibited multiple integrins, specifically α_2b_β_3_ (GP IIb/IIIa), α_5_β_1_, α_v_β_3_, α_v_β_1_, and α_v_β_5_, which act on platelet adhesion/aggregation. Saxatilin inhibited multiple integrins by acting on platelets, and was safe and effective in resolving thrombi in mice.

## Introduction

Intravenous (IV) administration of recombinant tissue plasminogen activator (rt-PA) is an effective treatment for ischemic strokes if administered within 4.5 hours of symptom onset [1,2]. However, more than two-thirds of patients fail to achieve successful recanalization after IV rt-PA treatment [3,4]. rt-PA also has neurotoxic effects [5]. To improve thrombolytic potency and to reduce the potential adverse effects of rt-PA, several new thrombolytic agents have been developed. They include variants of t-PA, and microplasmin, plasmin, and plasminogen activators from animal sources [6-9]. The goal of new thrombolytic drugs is enhanced fibrin specificity, extended plasma half-life, reduced inhibition by plasminogen activator inhibitor-1, and no neurotoxicity [10]. While new drugs target the fibrin component of thrombi, thrombi are formed by platelet-fibrinogen interaction. Resistance of platelet-rich thrombi to thrombolytic agents that target fibrin is a primary cause of thrombolysis failure. For this reason, treatments that target platelets might be useful since disaggregation of platelets from fibrin is a potential approach for dissolving thrombi [11,12].

Adhesion and aggregation of platelets are mediated by interactions of ligands with multiple integrins, including integrins α_2b_β_3_ (glycoprotein [GP] IIb/IIIa), α_2_β_1_, α_5_β_1_, and α_v_β_3_. Among these integrins, the GP IIb/IIIa receptor, which mediates the final common pathway of platelet aggregation by binding specifically to fibrinogen [13], is the main target of drugs developed to act against platelets. Several platelet GP IIb/IIIa receptor antagonists have been developed, including the Fab fragment of a human-mouse chimeric antibody against GP IIb/IIIa (abciximab), nonpeptide analogs of an Arg-Gly-Asp (RGD) peptide (tirofiban and lamifiban), and a cyclic heptapeptide disintegrin containing a Lys-Gly-Asp (KGD) motif (eptifibatide) [14-16]. These GP IIb/IIIa antagonists have been effective at resolving thrombi by dethrombotic mechanisms (disaggregation of platelets bound to fibrinogen) in selected patients with acute coronary syndrome or stroke [14,15,17-19].

Saxatilin, a novel disintegrin purified and cloned from the venom of the Korean snake *Gloydius saxatilis*, has the tripeptide sequence RGD [20,21]. Saxatilin has a molecular mass of 7712 Da, and strongly inhibits platelet activation [22] and platelet aggregation [21]. Considering the known effects of GP IIb/IIIa receptor inhibitors on thrombus resolution, saxatilin might possess thrombolytic effects. In this study, the effect of IV saxatilin on thrombus resolution was examined using a ferric chloride (FeCl_3_)-induced arterial thrombosis model in mice and platelet aggregometry [23,24]. We also introduced novel analytic measures to quantitate thrombolytic effects using FeCl_3_-induced arterial thrombosis model and platelet aggregometry. 

## Materials and Methods

### Ethics Statement

All animal procedures were reviewed and approved by the Institutional Animal Care and Use Committee (IACUC) of Yonsei University College of Medicine (approval number: 2010-0268) and were performed in strict accordance with the Association for Assessment and Accreditation of Laboratory Animal Care (AAALAC).

### Experimental animals and FeCl_3_-induced carotid artery thrombosis

Eight-week-old male Institute of Cancer Research (ICR) mice weighing 32-34 g were used. Animals were housed in a temperature-controlled animal facility under a 12/12 hours reversed light and dark cycle, in a plastic cage with soft bedding, and given with a free access to food and water. For operative procedures, animals were anesthetized with 5% isoflurane in a mixture of 70% N_2_O and 30% O_2_. Anesthesia was maintained with 2% isoflurane. During operative procedures, body temperature was monitored continuously with a rectal probe and maintained at 37.0 ± 0.2°C using a homeothermic blanket control unit and a heating pad (Harvard Apparatus, Holliston, MA, USA). A FeCl_3_-induced carotid thrombosis model was used to test the thrombolytic activity of saxatilin *in vivo*. A midline cervical incision was made, and the left common carotid artery was carefully dissected under a surgical microscope. An ultrasonic Doppler ﬂow probe (MA0.7PSB; Transonic Instruments, Ithaca, NY, USA) was placed around midportion of the common carotid artery (CCA). Carotid blood flow was obtained with a Transonic TS420 Blood Flow Meter (Transonic Instruments, Ithaca, NY, USA) and an iWorx IX-304T data acquisition system (iWorx Systems, Inc., Dover, NH). Computer-based analysis by iWorx Labscribe 2 software (version 2.045000) was performed to minimize any bias when assessing results. CCA baseline flow was measured for 5 minutes. Oxidative vascular injury with chemical stress was induced by placing a filter paper (700 μm × 500 μm) saturated with 50% FeCl_3_ (F2877; Sigma-Aldrich Inc., St. Louis, MO, USA) on the adventitial surface of the midpoint of the exposed CCA for 5 minutes. After removing the filter paper, the CCA was washed with normal saline and blood flow was recorded. Thrombus formation and arterial occlusion were determined by decreased blood flow, and complete occlusion was deﬁned as absence of blood flow for 10 minutes.

### Determination of thrombotic occlusion and measurement of thrombus size

Consistency of the mouse model for thrombus formation and size was assessed. Ten minutes after complete occlusion, injured CCA segments were excised, immediately immersed in 4% paraformaldehyde for fixation, and embedded in paraffin for histological analysis. Paraffin blocks were consecutively sectioned longitudinally into 3 μm slices. Sectioned slices were mounted on glass slides and stained with hematoxylin and eosin. Thrombus size (longitudinal length and area) for each animal was determined using a light microscope (Axio Imager.D2; Carl Zeiss Microimaging, Oberkochen, Germany) and Zeiss AxioVision software (AxioVs40 V 4.8.1.0; Carl Zeiss imaging Solution) for a slice representing the largest part of the thrombus.

### Electron microscopic examination

For transmission electron microscopy (TEM), arteries were immediately fixed with Karnovsky solution (pH 7.4, 2% glutaraldehyde, 2% paraformaldehyde, 0.5% CaCl_2_) overnight at 4°C, washed in 0.1 M phosphate buffer (pH 7.4) and post-fixed in 1% osmium tetroxide in the same buffer for 2 hours. Specimens were then dehydrated through a series of ascending ethanol concentrations, exchanged through propylene oxide, and incubated with a 1:1 mixture of EPON (EPON 812, MNA, DDSA, DMP 30) and propylene oxide for 18 hours, embedded in an EM oven, and trimmed. Subsequently, 0.25 μm semi-thin sections were stained with toluidine blue and observed under a light microscope to determine the FeCl_3_-damaged region. After retrimming, ultrathin sections (80 nm) were obtained by ultramicrotome (Ultracut UCT, Leica, Austria) with a diamond knife, double stained with uranyl acetate and lead citrate, and examined by TEM (JEOL-1011; JEOL, Japan) at 80 kV [25].

For scanning electron microscopy (SEM), specimens were fixed and dehydrated as described above, replaced with isoamyl acetate, dried in a critical point dryer (HCP-2; Hitachi Co., Tokyo, Japan), coated with a thin layer of gold (100 nm) in an ion coater (IB-3; Eiko engineering, Ibaraki, Japan), and examined with by field-emission SEM (S-800; Hitachi Co., Tokyo, Japan) at 20 kV. 

### Selection of optimal FeCl_3_ concentration

To determine the optimal condition of the FeCl_3_-induced arterial thrombosis model for evaluation of thrombolytic effects, 10%, 20%, 30%, 40%, and 50% (w/v) concentrations of the FeCl_3_ were tested using five mice for each test concentration. Carotid blood flow was measured continuously for 150 minutes from complete occlusion.

### Intravenous thrombolysis using saxatilin

Expression and mass production of saxatilin have been described [20,26]. Ten minutes after CCA occlusion, IV administration of saxatilin was performed via the left femoral vein with an infusion pump (KDS100 syringe pump; KD Scientific, Holliston, MA, USA) connected to PE-10 tubing. Carotid blood flow was continuously monitored for 2 hours from the initial time of injection.

#### Dose response to saxatilin

To evaluate the dose response to saxatilin, animals were randomly divided into seven groups, with five mice in each group: normal saline (control group), 1, 1.75, 2.5, 3.75, 5.0, or 10.0 mg/kg saxatilin. Ten percent of the dose was administered by IV bolus, and the rest was infused continuously for 60 minutes.

#### Methods for administering saxatilin

The thrombolytic effects of saxatilin were assessed after different administration methods. A total dose of 5 mg/kg of saxatilin was used in each animal, and animals were divided into the following four groups with five mice in each group: 1) bolus injection of total dose (5 mg/kg) at 10 minutes after occlusion; 2) double bolus injection of saxatilin with a half dose (2.5 mg/kg) of saxatilin at 10 minutes after occlusion and 60 minutes after the first bolus injection; 3) half-dose bolus injection (2.5 mg/kg) at 10 minutes after occlusion, then continuous infusion for 60 minutes of the remaining dose; or 4) bolus injection of 10% of the total dose (0.5 mg/kg) at 10 minutes after occlusion, and then continuous infusion of the remaining dose (4.5 mg/kg) for 60 minutes. 

### Assessment of recanalization

The presence and degree of recanalization were assessed by measuring blood flow. Immediately after 2 hours of blood-flow monitoring, the CCA was removed from all mice, fixed with 4% paraformaldehyde solution, and embedded in paraffin for histological examination. Paraffin blocks were consecutively sectioned in a transverse direction into 3 μm slices, mounted on glass slides, and stained with hematoxylin and eosin.

#### Dose response to saxatilin

Carotid blood flow was determined by calculating the area under time-flow curves. All measured values were standardized by the minimum blood flow of each animal to avoid differences from variations in physiological condition between animals. Thrombolytic effects were calculated as described below and expressed as percent of mean control baseline blood flow: (mean blood flow during 2 hours of monitoring/mean baseline blood flow) × 100. Mean values of each group in the dose-response study were calculated and graphed on a standard thrombolytic activity curve (mean ± SD).

#### Thrombolytic effects over time

Average blood flow was calculated for each animal each minute to achieve a representative time-dependent pattern during monitoring. Values for mean and standard deviation of all animals in each group were calculated, and temporal changes are shown as continuous bar graphs.

#### Time to recanalization

Time from administration of saxatilin to effective recanalization was assessed. Effective recanalization was defined as restoration of blood flow to at least 50% of the baseline level, maintained for longer than 30 minutes.

### Mechanism of action of saxatilin

#### Inhibition on platelet aggregation using platelet aggregometry

Blood (900 μl) was drawn by cardiac puncture from five mice anesthetized with isoflurane into a syringe containing 100 μl of 150 USP sodium heparin solution, resulting in a final heparin concentration of 15 USP/ml. A total of 500 μl of heparinized whole blood was mixed with the same volume of normal saline. Saxatilin concentrations were adjusted by dilution with normal saline, and 0.1, 1, or 2 μg of saxatilin against adenosine diphosphate (ADP) and 5, 50, or 100 μg of saxatilin against collagen were added into each test cuvette and preincubated with magnetic stirring at 37°C for 5 minutes. The same volume of normal saline was added as a control. Agonists for platelet aggregometry were 20 µM ADP or 5 μg/ml of collagen (Chronolog Corporation, Havertown, PA). Platelet aggregation activity was measured with an impedance method in a platelet aggregometer (Chronolog 700; Chronolog Corporation, Havertown, PA). 

#### Disaggregation of preformed platelet aggregated

To test saxatilin effects on preformed platelet aggregates, 10, 50, and 250 μg of saxatilin or the same volume of normal saline as a control were added to cuvettes after maximal aggregation in response to 20 μM ADP or 5 μg/ml of collagen. Platelet disaggregation effects were calculated as percentage of restoration to baseline levels. 

#### Fibrin/fibrinogen zymography

Fibrin/fibrinogen zymography was performed to determine fibrinolytic activity. Fibrinogen gels were prepared using 12% sodium dodecyl sulfate (SDS)-polyacrylamide gels containing 1.2% fibrinogen (Hyphen Biomed, Neuville-sur-Oise, France) and 0.1 NIH unit/ml of thrombin (Hyphen Biomed, Neuville-sur-Oise, France). A total of 100 μg saxatilin or 3 ng rt-PA (Actilyse; Boehringer Ingelheim, Ingelheim, Germany) with an equal volume of sample buffer (80 mM Tris-HCl, pH 6.8, 4% SDS, 10% glycerol, 0.01% bromophenol blue) was loaded into wells. Gels were rinsed in 150 ml of 2.5% Triton X-100 (15 minutes) and incubated with 250 ml reaction buffer (30 mM Tris, pH 7.4, 200 mM NaCl_2_, and 0.02% NaN_3_) for 12 hours at 37°C. Gels were stained with 0.1% amido black containing acetic acid, methanol, and distilled water (volume ratio 1:3:6) for 1 hour and destained by four washes with the same solution without amido black for 130 minutes. Gels were scanned using a flatbed scanner (ArtixScan F1; Microtek International Inc., Hsinchu, Taiwan) [25,27].

#### Binding affinity test for integrin receptors

To investigate the interaction of saxatilin with integrins, Fc-tagged recombinant protein was generated because there was no available antibody against saxatilin. The vector encoded the Fc region of human IgG1 (~230 amino acids). Interaction with integrins was evaluated using enzyme-linked immunosorbent assay (ELISA). Integrins for α_2b_β_3_, α_v_β_3_, α_5_β_1_, α_v_β_1_, α_v_β_5_, α_1_β_1_, and α_2_β_1_ (7148-A2, 3050-AV, 3230-A5, 6579-AV, 2528-AV, 7064-AB, and 5698-A2; R&D Systems, Minneapolis, MN, USA) (100 ng) were immobilized in 96-well immunoplates for 16 hours at 4°C. Each well was blocked with skim milk and washed three times with 0.05% PBS-T. Saxatilin-Fc (~100 nM) was added to wells and incubated for 2 hours at room temperature. After washing three times with PBS-T, anti-HuFc antibody conjugated to HRP (31413; Thermo Fisher Scientific Inc., Rockford, IL, USA) (1:2000) was added to wells and incubated for 1 hour at room temperature. Nonadherent antibodies were removed by washing three times with PBS-T. Substrate was o-phenylenediamine tablets solubilized in phosphatidylcholine buffer; 100 μl was added to each well and incubated for 10 minutes. Absorbance at 490 nm was read on a spectrophotometer. We determined the K_d_ values from the titration curve using the simple linearization method [28]. We can read relative saturation value (i) from the titration curve. According to the modified linearization method [29], the plot of 1/(1 - i) vs ligand concentration/*i* gives a straight line and evaluates the apparent dissociation constant. The slope of the plot is 1/K_d_.

### Comparison of thrombolytic effects of agents

We evaluated thrombolytic effects of other well-known plasminogen activators: rt-PA (Actilyse; Boehringer Ingelheim, Ingelheim, Germany), urokinase-type PA (u-PA) (Urokinase; Green Cross Corp., Yongin, Korea), and the GP IIb/IIIa receptor antagonists abciximab (ReoPro; Lilly Pharma Production GmbH & Co., Hamburg, Germany) and tirofiban (Aggrastat; Iroko Cardio Australia Pty Ltd, Sydney, Australia). Agents were administrated as 10% IV bolus injection with continuous infusion of the remaining 90% for 1 hour at 0.9, 1.8, 2.7, 4.8, 7.2, 9, or 18 mg/kg for rt-PA; 100, 500, 1000, 5000, 10,000, or 50,000 IU/kg for u-PA; 0.25, 0.5, 1, 2.5, 5, 10, 20, or 40 mg/kg for abciximab; and 0.5, 1.25, 2.5, 3.75, 5, or 10 mg/kg for tirofiban. An equal volume of normal saline was administered to control animals. 

### Half-life measurement of saxatilin in mice

To evaluate half-life of saxatilin *in vivo*, 8-week-old male ICR mice (31-36 g) were used. Saxatilin was conjugated with NHS-Rhodamine (Thermo Fisher Scientific Inc., Rockford, IL, USA) according to manufacturer’s instruction. NHS-Rhodamine was dissolved in 10 mg/ml of dimethylsulfoxide (Sigma-Aldrich Inc., St. Louis, MO, USA). Saxatilin and NHS-Rhodamine were mixed and incubated at room temperature for 1 hour, and then non-reacted NHS-Rhodamine was removed with PD-10 Desalting Columns (GE Healthcare Bio-Sciences Corp., Piscataway, NJ, USA). Rhodamine-labeled saxatilin was diluted in 0.5 mg/ml and was administered intravenously at 5 mg/kg. To isolate serum, blood was collected from the jugular vein at 0, 5, 10, 20, 40, and 80 minutes after administration of Rhodamine-labeled saxatilin. The blood was incubated at room temperature for 30 minutes in dark and serum was separated by centrifugation at 10,000 rpm for 10 minutes. The serum was placed in black 96-well microplate (Thermo Fisher Scientific Inc., Rockford, IL, USA). Fluorescence was acquired via LS 50B Fluorescence Spectrometer (PerkinElmer, Waltham, MA, USA).

### Thrombolytic effects on aged thrombus

Thrombosis was induced in the same manner as it was performed in the dose-response study. To avoid spontaneous recanalization, distal site of the CCA was ligated with Silkam 5-0 thread (Silkam, B Braun Aesculap, Tuttlingen, Germany) immediately after achieving complete occlusion. After 1, 3, 6, 12, or 24 hours, the CCA ligation was gently removed and then 5 mg/kg saxatilin was administered intravenously (10% bolus and then continuous infusion of the remaining dose for 60 minutes). Thrombus at a time of complete occlusion was used as control fresh thrombus. Randomly selected five mice were used in each group. To compare thrombolytic efficacy between saxatilin and rt-PA on aged thrombus, 9 mg/kg rt-PA was administered in the same way at 3 or 6 hours after complete occlusion.

### Safety assessment

#### Hematological assessment

To assess whether saxatilin administration causes thrombocytopenia or neutropenia, hematologic analyses were performed with blood collected from 5 mice after sham operation. Immediately after infusion of 5 mg/kg saxatilin or same volume of normal saline for 60 minutes, 1 ml of blood was collected in 10% EDTA via the lateral saphenous vein. Complete blood count (CBC) with differential counts was obtained using the automatic cell counter (MS9-5V; Melet Schloesing Laboratories, Cergy-Pontoise, France). 

#### Bleeding time assays

Randomly selected four mice were used in each group. Immediately after infusion of 5 mg/kg saxatilin or same volume of normal saline for 60 minutes, bleeding time was assessed by transection of the tail at 1 cm from the tail tip using a sharp scalpel. Blood was blotted every 15 seconds and the time period from tail transection to cessation of bleeding was defined as the bleeding time. Bleeding times exceeding 600 seconds were considered as 600 seconds.

### Statistical analysis

Statistical analyses were performed using SPSS (version 20.0, SPSS Inc., Chicago, IL, USA). Normality of distributions was verified using the Kolmogorov-Smirnov test. Differences among groups in dose response studies were compared with a one-way ANOVA test, followed by a post-hoc Tukey method. Differences between groups in hematological assessment were compared with a Mann-Whitney U test. Values were presented as a mean ± standard deviation (SD). P < 0.05 was considered significant.

## Results

### Arterial response to various FeCl_3_ concentrations

**Figure 1:**
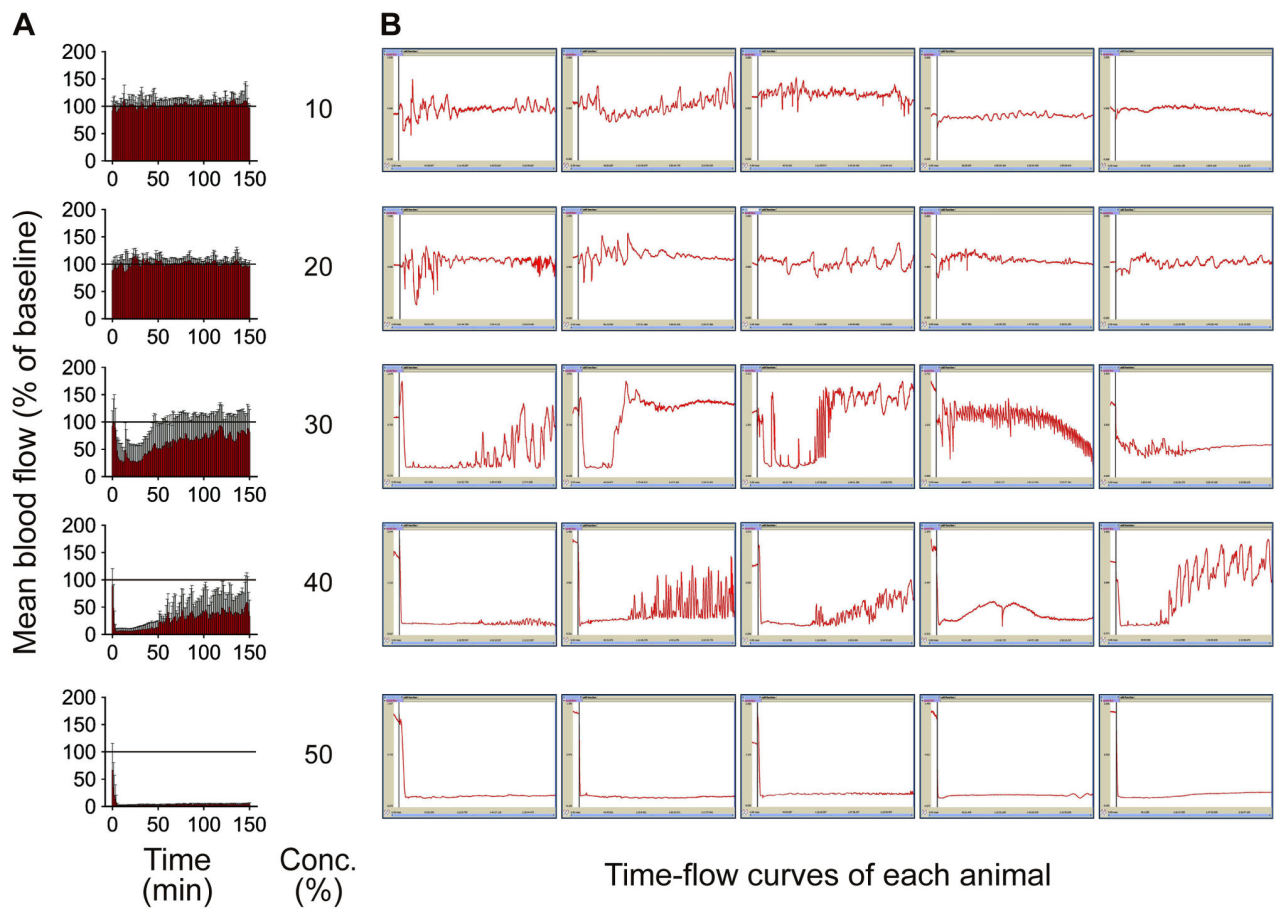
Arterial response to various concentrations (10-50%) of FeCl_3_. **A**: mean values of all animals in a group were calculated. Temporal changes are shown as continuous bar graphs (mean ± SD). **B**: Time-flow curves of all mice in each group. All mice by 50% FeCl_3_ achieved complete occlusion, which was maintained without spontaneous recanalization during period for 150 minutes of monitoring.

No complete occlusion was observed by 10% or 20% FeCl_3_. Two of the five mice by 30% and all of the five mice by 40% achieved complete occlusion, but all of them showed spontaneous recanalization during monitoring for 150 minutes except one mouse by 40%. All mice by 50% FeCl_3_ achieved complete occlusion, which was maintained without spontaneous recanalization during the monitoring (Figure 1). Therefore, 50% FeCl_3_ was used for the remaining experiments. 

### Consistency of the animal model

**Figure 2:**
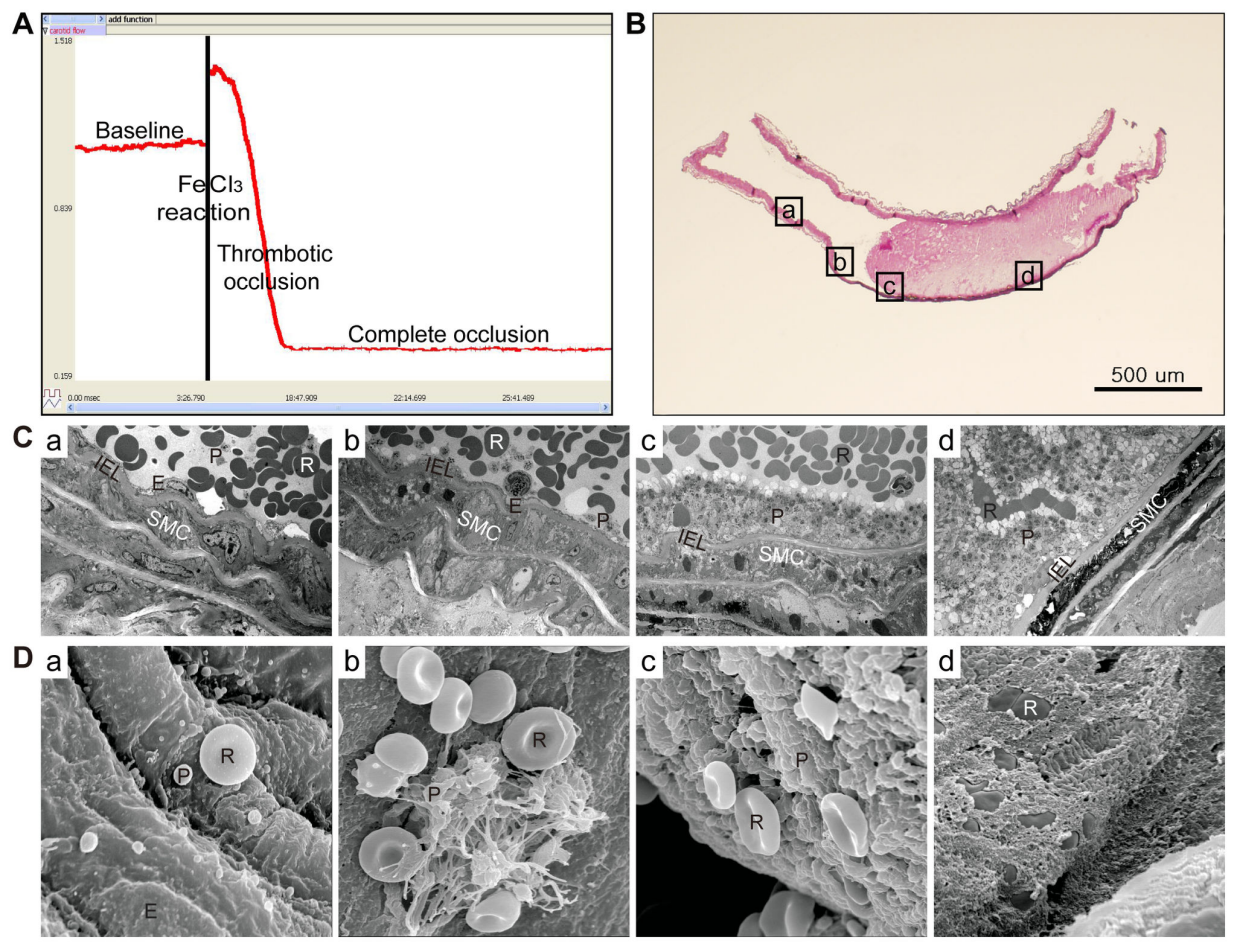
Reliability of the FeCl_3_-induced arterial thrombosis model. **A**: Blood flow was monitored using an ultrasonic Doppler ﬂow meter in the carotid artery. Representative time-flow curve from FeCl_3_-induced arterial thrombosis. Complete occlusion was defined as absence of blood flow for 10 minutes. **B**: Hematoxylin and eosin staining of the carotid artery with thrombotic occlusion after FeCl_3_ treatment. Original magnification ×100. **C**: Transmission electron microscopy. **D**: Scanning electron microscopy. Boxes in Figure 2B indicate sites of electron microscopy images. **a**) Region farthest from the site of FeCl_3_ application with intact vascular structure and normally shaped erythrocytes and discoid platelets. **b**) Mildly damaged region. Empty endothelial cells with loss of organelles, activated and aggregated platelets, and platelets adhering to the luminal surface. **c**) Thrombus border region with no intact endothelial cells. Activated and aggregated platelets firmly adhered to the damaged region of the luminal surface. **d**) Severely damaged region with artery completely occluded by a platelet-rich thrombus containing several erythrocytes. FeCl_3_-induced damage caused loss of vascular undulation, but the internal elastic lamina was intact even in the severely damaged region. R: erythrocyte, P: platelet, E: endothelial cell, SMC: smooth muscle cell, IEL: internal elastic lamina. Original magnification ×2,000 (TEM), ×5,000 (SEM).

After 5 minutes of FeCl_3_, CCA blood flow was rapidly and consistently reduced to nearly zero in all five animals examined (Figure 2A). CCA thrombotic occlusion was demonstrated by histologic examination (Figure 2B). Thrombus sizes were similar among the animals (length: 1.139 ± 0.091 mm; area: 0.305 ± 0.055 mm^2^). 

### Electron microscope features of the carotid artery and thrombus after FeCl_3_

The regions farthest from where FeCl_3_ was applied showed relatively normal vascular structure, with normally shaped endothelial cells, smooth muscle cells, erythrocytes, and discoid platelets (Figures 2Ca, Da). Endothelial cells appeared empty with a loss of organelles. Some platelets were activated and aggregated and adhered to the luminal surface in mildly damaged regions (Figures 2Cb, Db). At the thrombus border, activated and aggregated platelets adhered to the damaged luminal surface, and no intact endothelial cells were observed (Figures 2Cc, Dc). In severely damaged regions, platelet-rich thrombi containing erythrocytes completely occluded the vascular lumen (Figures 2Cd, Dd). FeCl_3_ treatment caused a loss of vascular undulation with a flattened and disconnected arrangement of smooth muscle cells. The internal elastic lamina remained intact even in severely damaged regions.

### Dose-dependent thrombolytic effects of saxatilin

**Figure 3:**
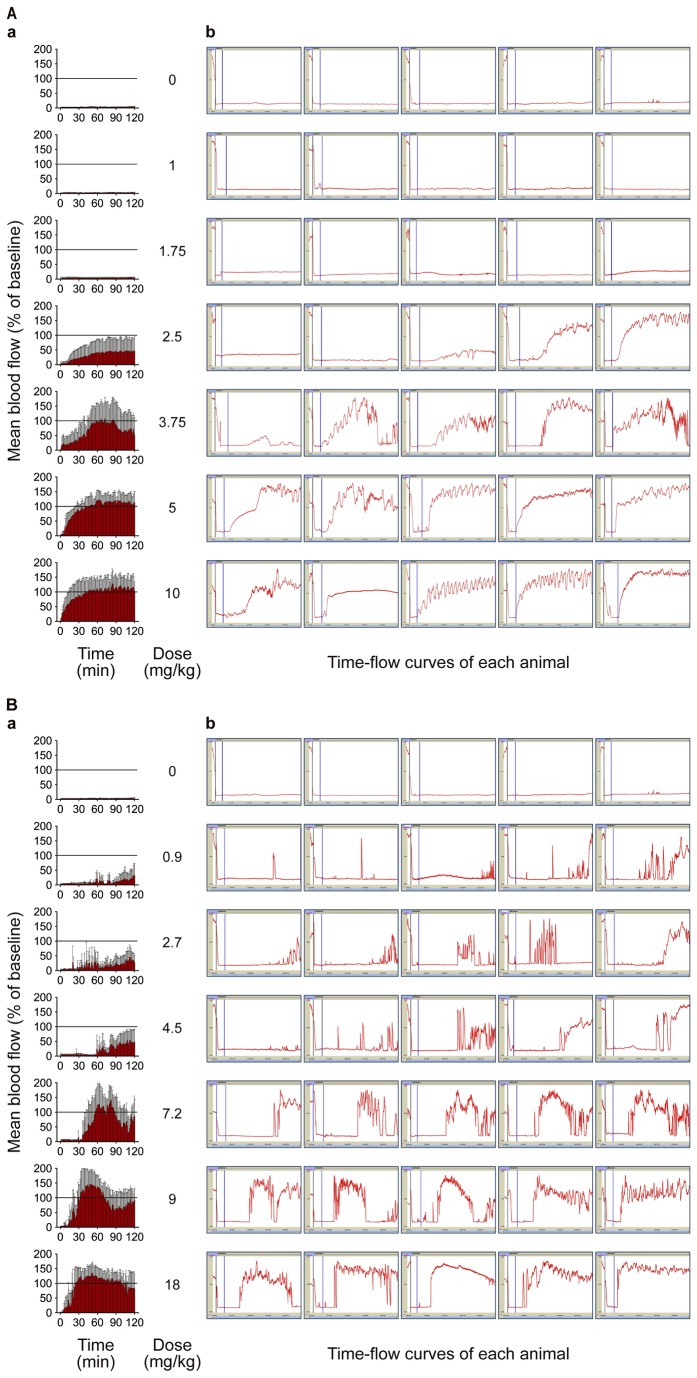
Dose-dependent thrombolytic effects of saxatilin (A) and rt-PA (B). **a**) Mean values of all animals in a group were calculated. Temporal changes are shown as continuous bar graphs (mean ± SD). **b**) Time-flow curves of all mice in each group. As the dose of saxatilin or rt-PA increases, the time to recanalization and the frequency of reocclusion decreases. Saxatilin showed relatively short time to recanalization and low frequency of reocclusion compared with rt-PA.

Saxatilin treatment did not cause any notable changes in blood flow at a dose of 1 mg/kg (2.36 ± 0.78%) or 1.75 mg/kg (5.14 ± 2.92%) compared to the normal saline group (2.42 ± 1.07%) (Figure 3A, 9A). Blood flow was restored at 2.5 mg/kg (32.50 ± 33.70%) and increased in a dose-dependent manner. At 3.75 mg/kg saxatilin, blood flow was significantly increased compared to the normal saline control (60.50 ± 38.78%, p = 0.019), and 5 mg/kg restored blood flow to nearly baseline levels (94.50 ± 20.47%). No significant differences were observed between 5 and 10 mg/kg of saxatilin (94.94 ± 39.05%, p > 0.999).

### Effect of saxatilin by method of administration

**Figure 4:**
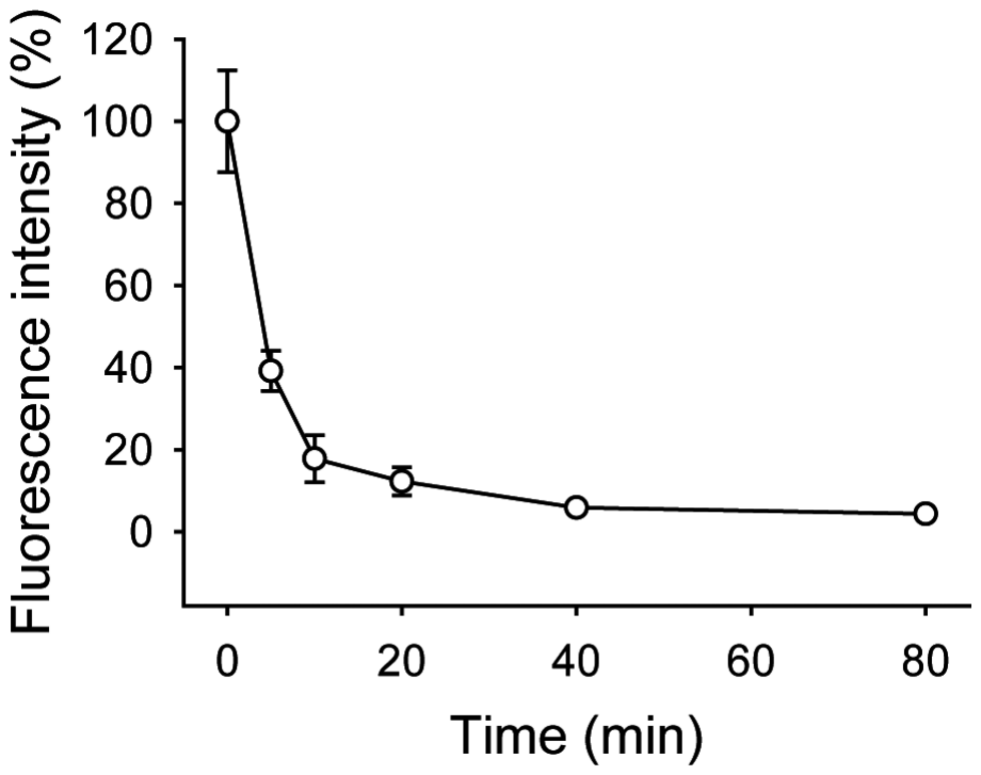
Half-life of saxatilin in mice. Fluorescence was measured from sera isolated at the indicated time-points (0, 5, 10, 20, 40, or 80 minutes) after administration of Rhodamine-labeled saxatilin. Half-life of saxatilin was 4.1 minutes in mice. Values are presented as a mean ± standard deviation.

Half-life of saxatilin, which was measured using saxatilin labeled with NHS-Rhodamine, was 4.1 minutes (Figure 4). 

**Figure 5:**
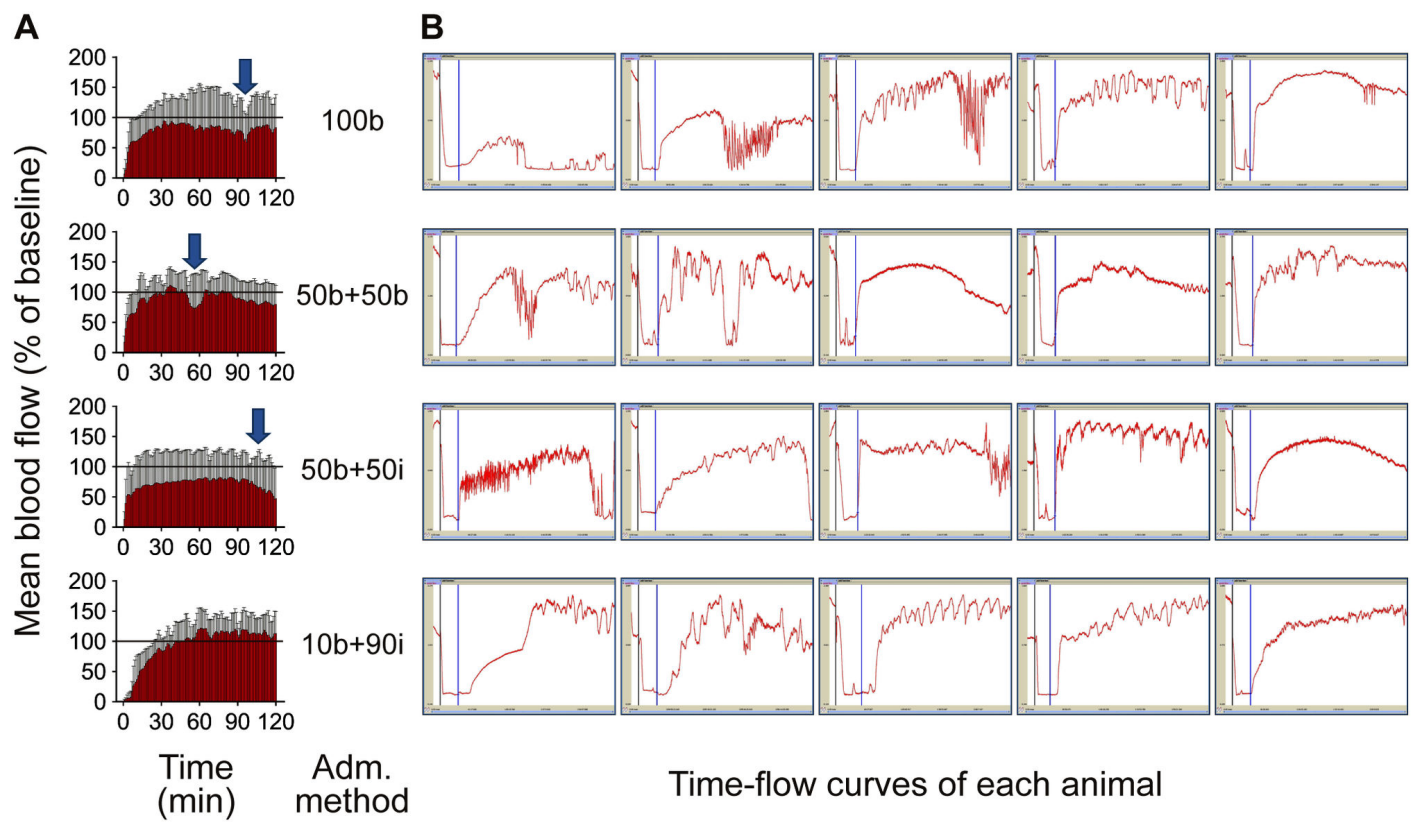
Thrombolytic effect of saxatilin according to administration method. **A**: Blood flow patterns after saxatilin. Abrupt reocclusion in mice after bolus injection of total dose or 50% of total dose. No reocclusion with bolus injection of 10% total dose with continuous infusion of remaining dose. **B**: Time-flow curves of all mice in each group. *b*, bolus injection; *i*, continuous infusion. Arrows indicate reocclusion development.

Saxatilin at 5 mg/kg was effective in dose-response studies, so this dosage was used to determine the optimal method of intravenous administration of saxatilin (Figure 5). Mean percentages of blood flow compared to baseline blood flow were 77.01 ± 46.11% in a group that received saxatilin by a bolus injection of the total dose; 85.23 ± 29.95% in a group that received a double bolus injection with half of a dose; 80.72 ± 30.13% in a group that received a half-dose bolus injection with continuous infusion of the remaining dose; and 94.50 ± 20.47% in a group that received a bolus injection of 10% of the total dose with continuous infusion of the remaining dose. 

Decreased thrombolytic effects were observed at different times according to administration method. Abrupt reocclusion was observed approximately 50 minutes after the first bolus injection in mice treated with double bolus injection, approximately 100 minutes after a total dose bolus injection, and approximately 110 minutes after a half-dose bolus injection with continuous infusion of the remaining half dose. Reocclusion was not observed with a bolus injection of 10% of the total dose and continuous infusion of the remaining dose (Figure 5).

### Time to effective recanalization by saxatilin

**Figure 6:**
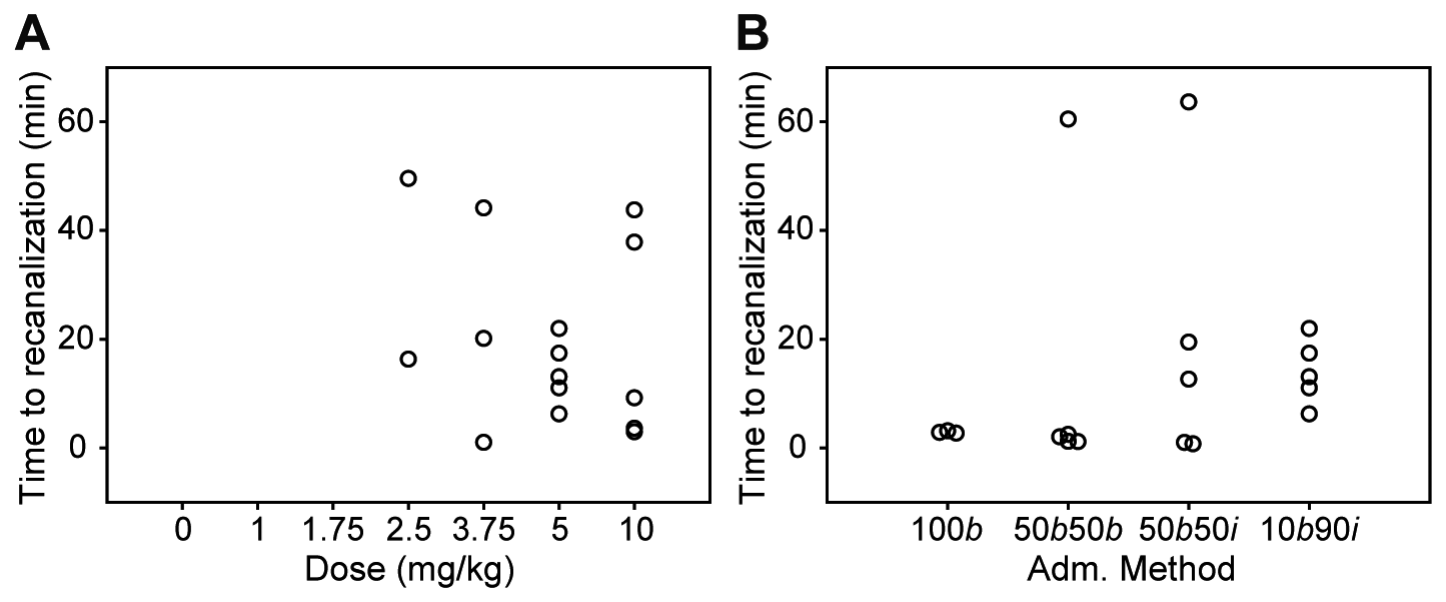
Time to effective recanalization. Effective recanalization was defined as restoration of blood flow to at least 50% of baseline levels, maintained for longer than 30 minutes. **A**: Time to effective recanalization by administration dose. No effective recanalization at 0, 1, or 1.75 mg/kg. Two mice in the 2.5 mg/kg and three mice in the 3.75 mg/kg groups and mice with 5 or 10 mg/kg showed effective recanalization. **B**: Time to effective recanalization by administration method. All mice except two administered a bolus injection of the total dose showed effective recanalization. *b*, bolus injection; *i*, continuous infusion.

Effective recanalization was not observed in mice treated with normal saline, 1 mg/kg saxatilin, or 1.75 mg/kg saxatilin. Only two of five mice treated with 2.5 mg/kg of saxatilin and three of five mice treated with 3.75 mg/kg of saxatilin showed effective recanalization. Effective recanalization was observed in all mice treated with saxatilin at 5 or 10 mg/kg (Figure 6A). The time to effective recanalization was 32.92 ± 23.52 minutes in mice treated with 2.5 mg/kg saxatilin, 21.75 ± 21.62 minutes in mice treated with 3.75 mg/kg, 13.92 ± 6.02 min in mice treated with 5 mg/kg, and 19.46 ± 19.75 minutes in mice treated with 10 mg/kg of saxatilin (Figure 6A). 

Effective recanalization was also evaluated according to saxatilin administration method. All mice treated with 5 mg/kg saxatilin achieved effective recanalization except for two mice administered a bolus injection of the total dose. Time to effective recanalization was 2.86 ± 0.22 minutes in mice treated with a bolus injection of the total dose, 13.44 ± 26.31 minutes in mice that received a double bolus injection, 19.48 ± 25.94 minutes in mice that received a half-dose bolus injection followed by continuous infusion of the other half dose, and 13.92 ± 6.02 minutes in mice treated with a bolus injection of 10% of the total dose with continuous infusion of the remaining dose (Figure 6B). 

### Effects of saxatilin on platelet aggregation

**Figure 7:**
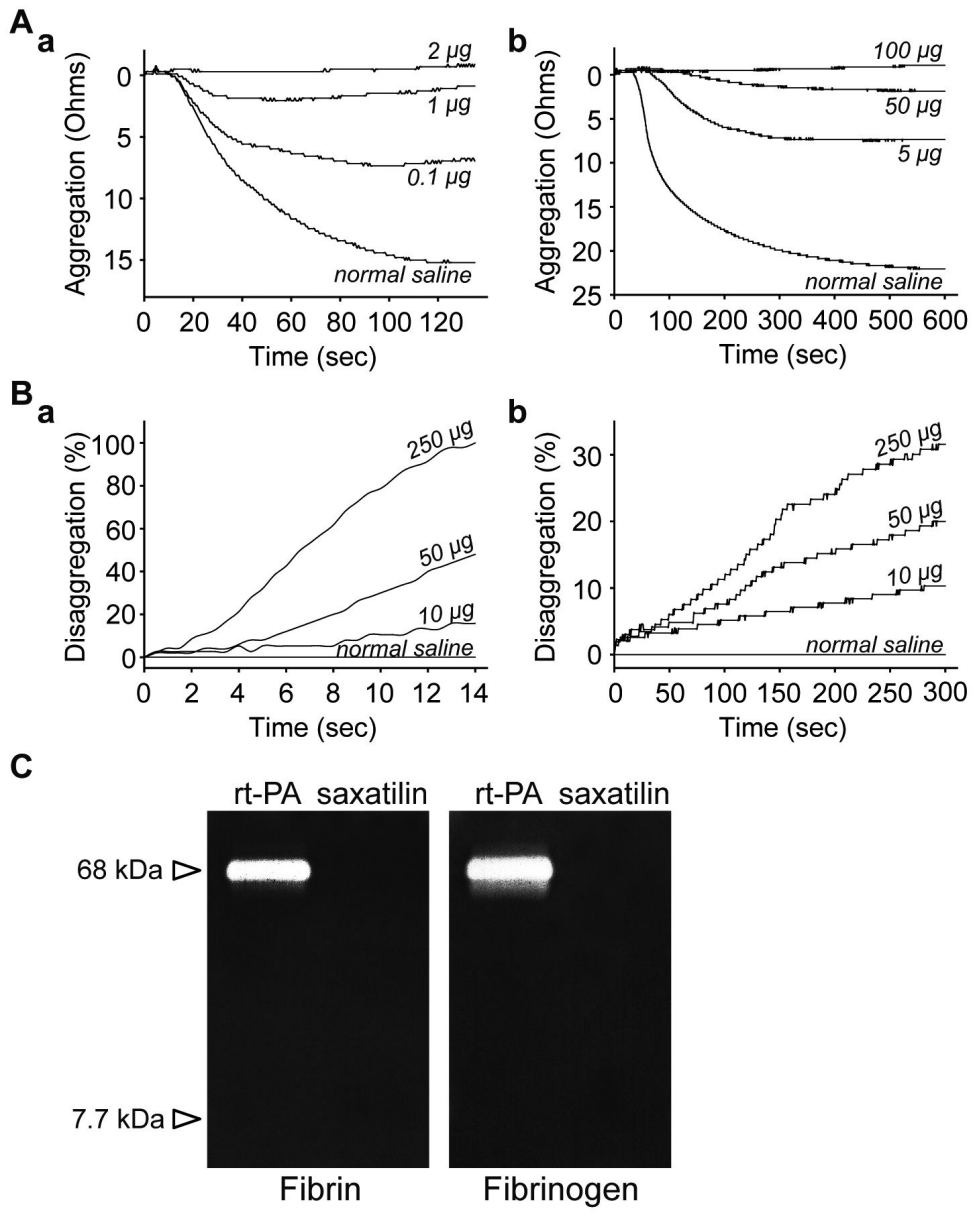
Mechanism of thrombus dissolution by saxatilin. **A**: Inhibitory effect of platelet aggregation by saxatilin. **a**) Inhibition in response to 20 μM ADP or **b)** 5 μg/ml collagen. Platelet aggregometry shows dose-dependent inhibition of platelet aggregation. **B**: Effect of saxatilin on platelet disaggregation. **a**) Saxatilin effect on a preformed thrombus induced by 20 μM ADP, or **b**) 5 μg/ml collagen. Graphs show dose-dependent disaggregation by saxatilin on the preformed thrombus. **C**: Fibrin/fibrinogen zymography with rt-PA bands. No band was seen after saxatilin loading.

Platelet aggregometry showed that saxatilin has a strong inhibitory effect on platelet aggregation (Figure 7A). Saxatilin inhibited 50% of platelet aggregation at 0.1 μg/ml (8 ohms), 87.5% at 1 μg/ml (7 ohms), and 100% at 2 μg/ml (0 ohm) in response to 20 μM ADP (maximal control aggregation was 16 ohms) (Figure 7Aa). Saxatilin inhibited 63.64% of platelet aggregation at 5 μg/ml (8 ohms), 90.91% at 50 μg/ml (2 ohms), and 95.45% at 100 μg/ml (1 ohm) in response to 5 μg/ml collagen (maximal control aggregation was 22 ohms) (Figure 7Ab). 

Experiments using preformed thrombi showed dose-dependent disaggregating effects of saxatilin (Figure 7B). ADP-induced platelet aggregates were resolved rapidly. At 14 seconds after addition, impedance was restored by 15.79% at 10 μg/ml, 48% at 50 μg/ml, and to basal levels (100%) at 250 μg/ml saxatilin (Figure 7Ba). The response to the collagen-induced platelet aggregates was relatively slow. At 300 seconds after addition, impedance was restored by 10.32% at 10 μg/ml, by 20% at 50 μg/ml, and by 31.58% at 250 μg/ml saxatilin (Figure 7Bb). 

### Fibrinolytic effect of saxatilin

Both fibrin and fibrinogen zymograms showed clear bands of rt-PA. However, saxatilin did not demonstrate a clear fibrinolysis band, which suggested that saxatilin had no fibrinolytic activity (Figure 7C).

### Integrin binding assay

**Figure 8:**
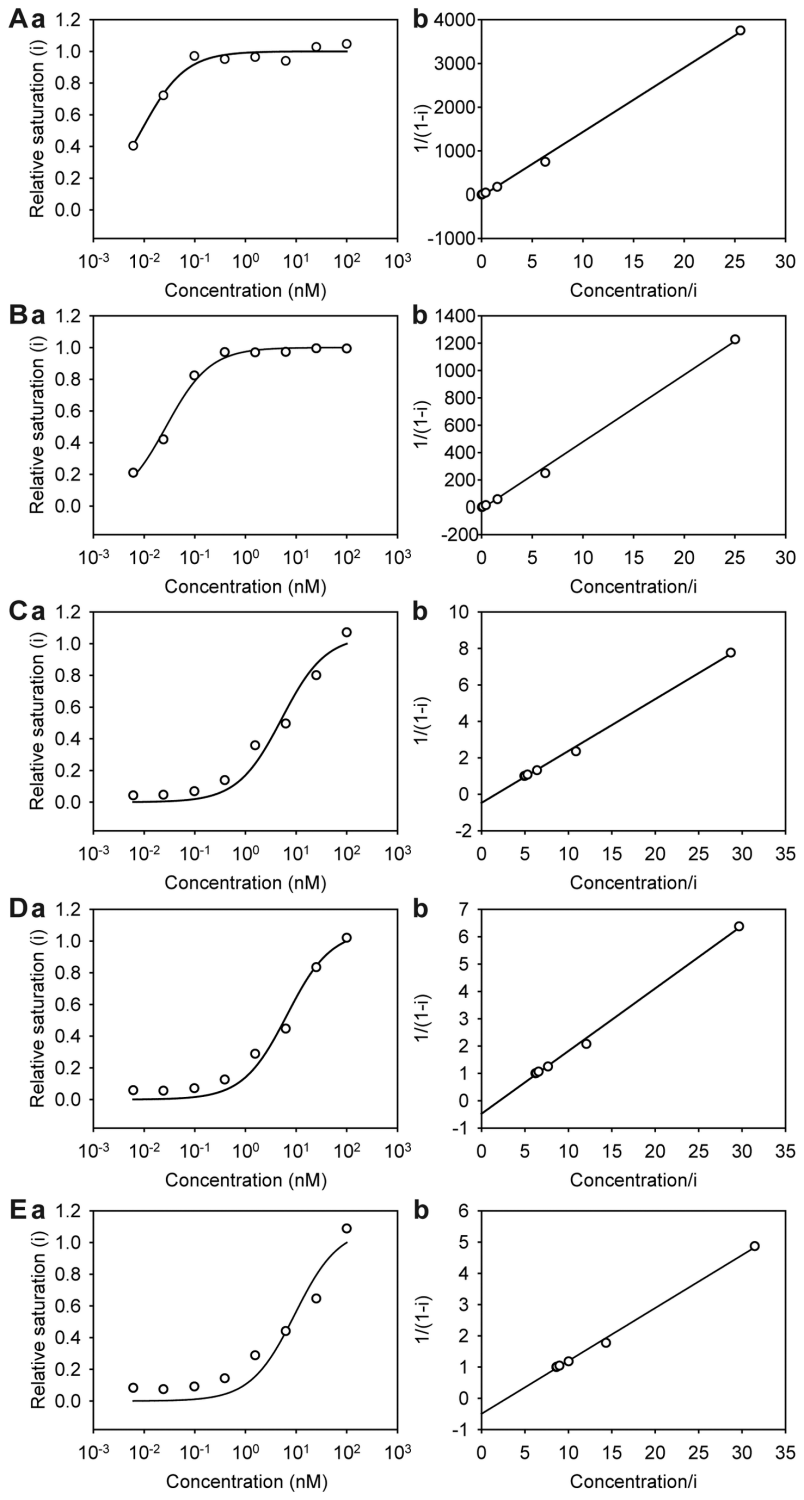
Binding affinity of saxatilin-Fc for integrins α_2b_β_3_ (A), α_v_β_3_ (B), α_v_β_5_ (C), α_5_β_1_ (D), and α_v_β_1_ (E). **a**) Titration curves obtained from ELISA. The relative titration, *i*, is displayed as the function of saxatilin-Fc concentration. **b**) Plot of data (a) according to equation 1/(1 - *i*) = (saxatilin-Fc concentration/*i*)/(K_d_) - b. The parameter of the straight line, the slope (1/K_d_) is shown in the figure.

We investigated the binding affinity of saxatilin-Fc to various integrins (Figure 8). Saxatilin-Fc bound to integrin α_2b_β_3_ with the highest affinity (K_d_ = 6.8 × 10^-12^ M). Saxatilin-Fc also showed high affinity for integrins α_v_β_3_ (K_d_ = 2.0 × 10^-11^ M), α_v_β_5_ (K_d_ = 3.5 × 10^-9^ M), α_5_β_1_ (K_d_ = 4.4 × 10^-9^ M), and α_v_β_1_ (K_d_ = 5.9 × 10^-9^ M). Saxatilin-Fc did not bind to integrins α_1_β_1_ or α_2_β_1_.

### Dose-dependent thrombolytic effects of rt-PA, u-PA, abciximab, and tirofiban

**Figure 9:**
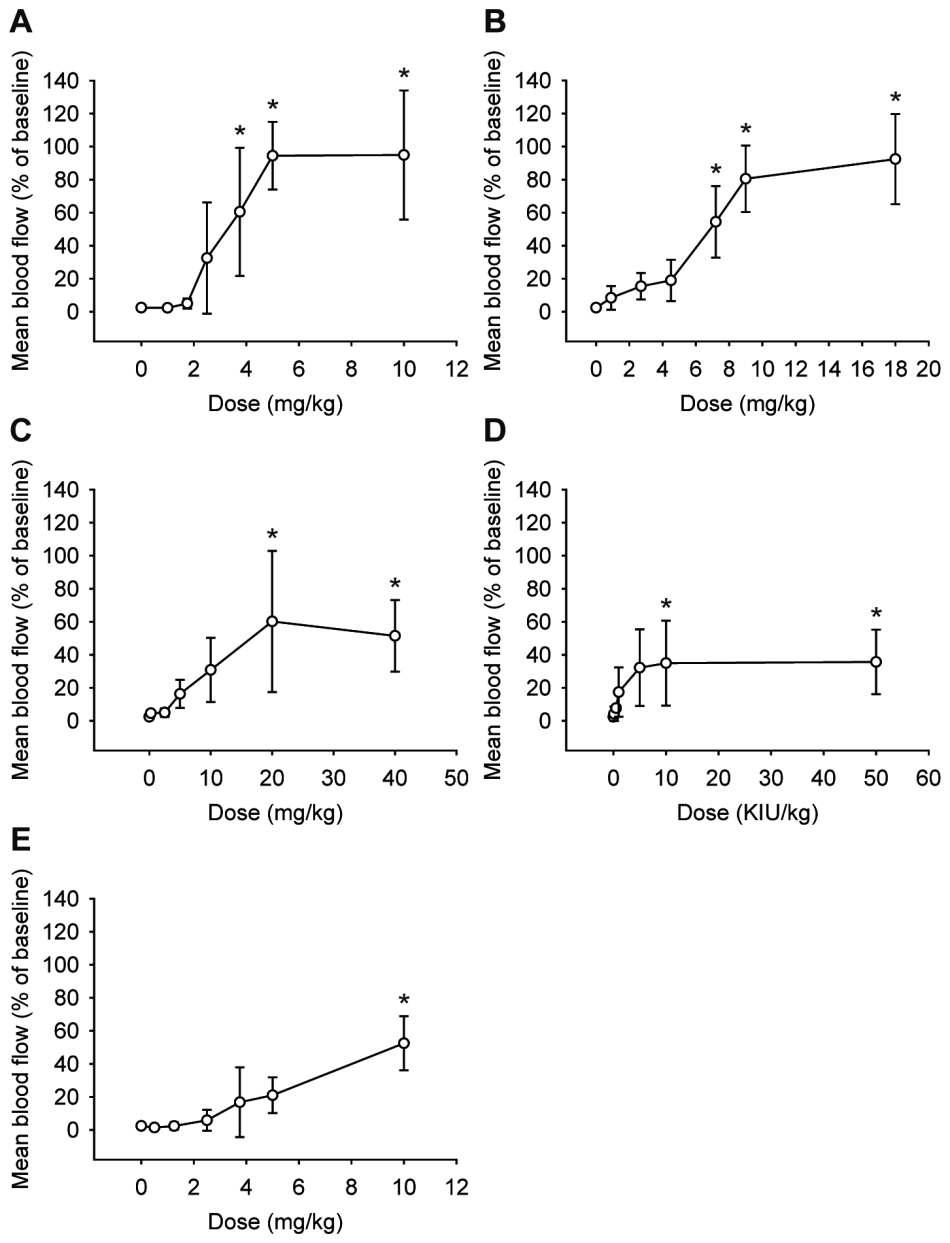
Dose-dependent thrombolytic effects of thrombolytic agents. Dose-response curves of saxatilin (**A**), rt-PA (**B**), u-PA (**C**), abciximab (**D**), and tirofiban (**E**). Saxatilin showed no notable changes until 1.75 mg/kg. Blood flow restored at 2.5 mg/kg, increased dose dependently, was significant at 3.75 mg/kg (p = 0.019), and was nearly baseline at 5 mg/kg; 5 vs. 10 mg/kg saxatilin (p > 0.999). Blood flow restoration was significant at rt-PA 7.2 mg/kg (54.45 ± 21.68%, p = 0.001), u-PA 10,000 IU/kg (34.96 ± 25.74%, p = 0.049), abciximab 20 mg/kg (60.19 ± 42.78%, p = 0.002), and tirofiban 10 mg/kg (52.47 ± 16.36%, p < 0.001).

Dose-dependent effects of thrombolytic agents were determined by calculating the area under time-flow curves (Figure 9). After rt-PA administration (Figure 3B, 9B), blood flow restoration increased in a dose-dependent manner and was significant at 7.2 mg/kg (54.45 ± 21.68%, p = 0.001) compared to the normal saline group (2.42 ± 1.07%). Blood flow was restored to nearly baseline levels at 9 mg/kg (80.52 ± 20.16%) and 18 mg/kg (92.44 ± 27.27%) of rt-PA. In contrast to rt-PA, the restoration of blood flow was about 35% of baseline after administration of u-PA (34.96 ± 25.74% at 10,000 IU/kg, and 35.68 ± 19.54% at 50,000 IU/kg) (Figure 9C). For abciximab, blood flow restoration was observed at 5 mg/kg (16.32 ± 8.52%), and increased in a dose-dependent manner. Blood flow restoration was significant compared to the normal saline group and maximal at 20 mg/kg (60.19 ± 42.78%, p = 0.002) (Figure 9D). After administration of tirofiban, blood flow restoration was significant compared to the normal saline group and maximal at a dose of 10 mg/kg (52.47 ± 16.36%, p < 0.001) (Figure 9E).

### Thrombolytic effects on the aged thrombus

**Figure 10:**
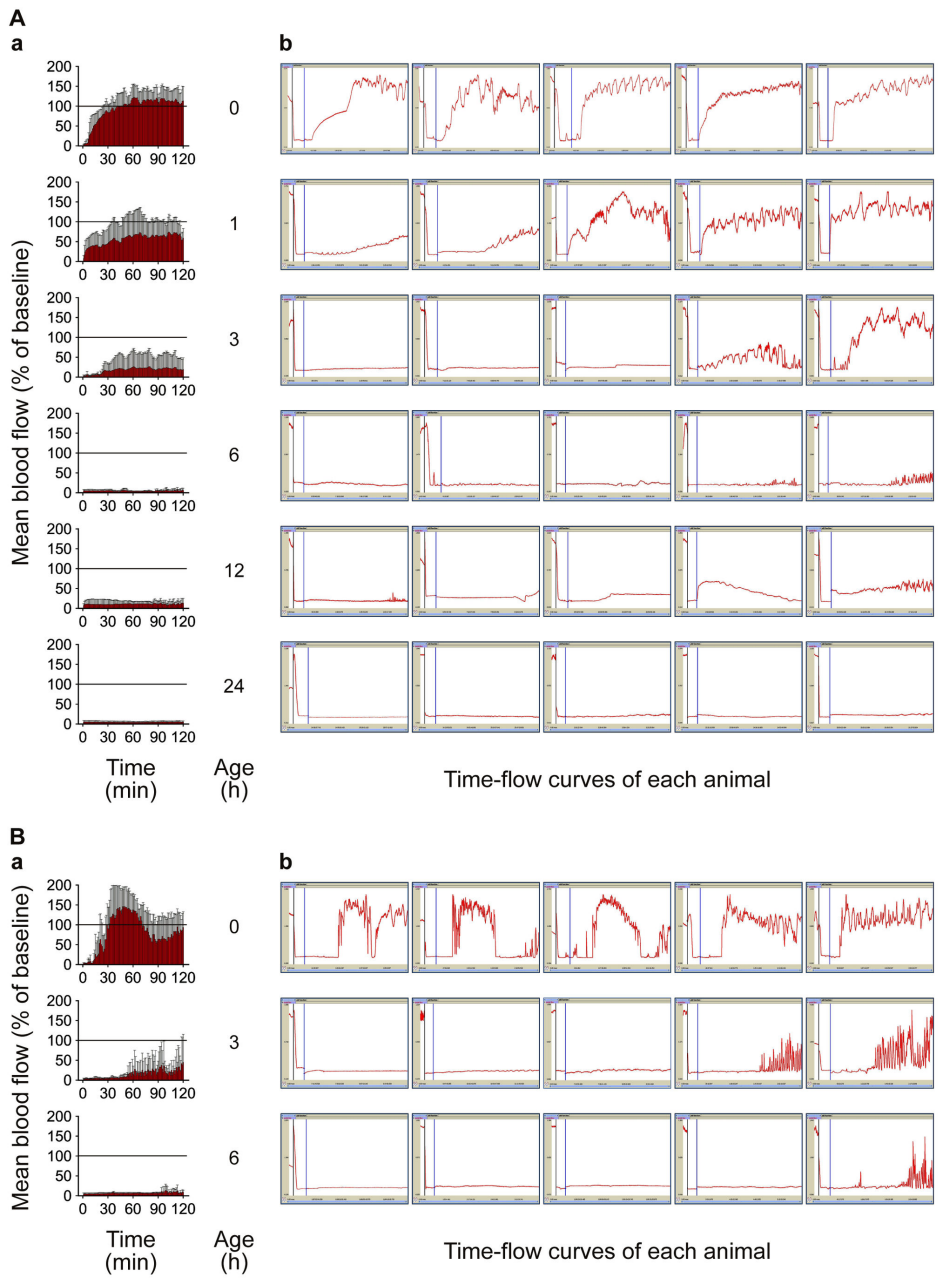
Thrombolytic effects of saxatilin (A) and rt-PA (B) on the aged thrombus. **a**) Mean values of all animals in a group were calculated. Temporal changes are shown as continuous bar graphs (mean ± SD). **b**) Time-flow curves of all mice in each group. As the thrombus age increases, the thrombolytic effect decreases.

Mean percentages of blood flow compared to baseline blood flow were 94.50 ± 20.47% in the group that received 5 mg/kg saxatilin at 1 hour; 17.71 ± 27.27% at 3 hours; 4.48 ± 1.37% at 6 hours; 10.67 ± 6.06% at 12 hours; and 4.51 ± 3.04% at 24 hours (Figure 10A). Mean percentages of blood flow compared to baseline blood flow were 14.26 ± 17.38% in a group that received 9 mg/kg rt-PA at 3 hours and 6.69 ± 1.66% at 6 hours (Figure 10B).

### Safety assessment

#### Mortality and bleeding after saxatilin treatment

Thrombolytic effects of saxatilin were evaluated using 71 animals. Two animals had bleeding at the cervical incision site, and one died of bleeding approximately 90 minutes after a bolus injection of saxatilin. The two animals with bleeding complications had received a single bolus dose of 5 mg/kg. None of the mice in the other groups showed bleeding complications. 

#### Hematologic assessment and bleeding time

No significant differences in hematologic parameters were observed between normal saline-infusion group and 5 mg/kg saxatilin-infusion group (Table 1). The tail bleeding time of normal saline-infusion group was 146.25 ± 14.36 seconds. All 5 mg/kg saxatilin-infused mice showed prolonged bleeding times exceeding 600 seconds.

**Table 1:** Effects of saxatilin on the hematologic parameters in mice.

	Control	Saxatilin	P-value
Red blood cell, ×10^6^/mm^3^	6.97 ± 1.33	7.23 ± 0.53	0.465
Hemoglobin, g/dl	11.15 ± 0.64	11.70 ± 0.57	0.346
Hematocrit, %	46.20 ± 9.05	43.90 ± 5.37	0.917
Platelet, ×10^3^/mm^3^	1114.00 ± 121.62	1132.00 ± 52.33	0.917
White blood cell, ×10^3^/mm^3^	3.41 ± 0.90	1.40 ± 0.40	0.754
Neutrophil, %	12.85 ± 14.35	13.25 ± 12.80	0.465
Lymphocyte, %	80.80 ± 16.55	79.55 ± 15.77	0.465
Monocyte, %	4.70 ± 3.11	4.85 ± 2.90	0.293
Eosinophil, %	0.90 ± 0.85	2.00 ± 0.28	0.917
Basophil, %	0.75 ± 0.07	0.35 ± 0.35	0.112

Values are presented as a mean ± standard deviation.

## Discussion

We demonstrated that saxatilin has strong thrombolytic effects *in vivo* and *in vitro*. The thrombolytic effects of saxatilin were dose dependent. Time to recanalization decreased with increasing saxatilin dosage. Reponses to saxatilin varied with administration method. Administration of the total dose in a bolus resulted in fast effective recanalization (approximately 3 minutes). However, reocclusion was frequently observed, and was also observed in mice treated with a bolus injection of 50% of the total dose. The regimen of a bolus injection of 10% of the total dose with continuous infusion of the remaining dose for 60 minutes resulted in a longer time to effective recanalization than treatment with a high-dose bolus injection. However, this regimen did not show reocclusion. These findings might result from the relatively short half-life of saxatilin, which was 4.1 minutes in mice.

The findings of this study indicated that saxatilin had thrombolytic effects on platelets but not fibrin/fibrinogen. Saxatilin is an RGD-containing protein. RGD-containing proteins from snake venom are characterized by strong conservation of the tripeptide RGD and by disulfide bond arrangements. These structural features might be critical for potential inhibition of platelet adhesion/aggregation and antagonism of integrin-mediated adhesion [30]. In an integrin-binding assay, saxatilin bound to integrins α_2b_β_3,_ α_v_β_3_, α_5_β_1_, α_v_β_1_, and α_v_β_5,_ with the highest affinity for α_2b_β_3_. Administration of GP IIb/IIIa antagonists not only prevents thrombus formation, but also disaggregates preformed fresh thrombi through competitive binding with the platelet GP IIb/IIIa receptor to the RGD domain of fibrin or fibrinogen [11]. Saxatilin lyses clots by dethrombosis and prevention of rethrombosis, similar to other GP IIb/IIIa antagonists. Saxatilin might have additive effects on dethrombosis and antithrombosis by acting against multiple integrins. α_2b_β_3_ does not act alone in mediating platelet adhesion/aggregation, but as part of a synergistic, multiple integrin-ligand association during platelet adhesion and aggregation [31]. In addition to α_2b_β_3,_ saxatilin showed high affinity for α_v_β_3_ and α_5_β_1_, which mediate platelet function along with α_2b_β_3_ [32]. Strong platelet adhesion, which is followed by initial interaction between GP1b and von Willebrand factor, is mediated by integrins such as α_5_β_1_ and α_2b_β_3_ [33]. Integrins α_5_β_1_ and α_2b_β_3_ share a fibrinogen-binding site [34]. Integrin α_5_β_1_ is involved in contraction of fibrin clots as well as platelet adhesion [35]. Integrin α_v_β_3_ is involved in thrombus formation by adhering not only to ligands for α_2b_β_3_ (i.e., von Willebrand factor, fibrinogen, fibronectin, vitronectin, osteopontin, and thrombospondin), but also to collagen [33,36]. Thus, maximal disaggregation of platelets from fibrinogen and inhibition of platelet adhesion/aggregation and thrombus growth might be achieved by blocking the effects of multiple integrins on platelets. These effects might be associated with the increased thrombolytic effects in our experiments compared to abciximab and tirofiban, which are well-known GP IIb/IIIa receptor antagonists.

Saxatilin appears to be safe because its effective dose was low and it rarely caused bleeding complications. In addition, saxatilin did not cause any notable changes in hematological parameters including platelet and neutrophil counts compared with control group. The most efficient dose in our study (5 mg/kg) was much lower than the known LD_50_ of saxatilin (400 mg/kg) in ICR mice [26]. In tail bleeding assays, 5 mg/kg saxatilin prolonged bleeding times. However, among the 71 mice in this study that received saxatilin for evaluation of thrombolytic effects by various dosage, administering methods, and thrombus ages, only two bled at the cervical incision site. These two mice received a single bolus of 5 mg/kg, the highest quantity administered in a single dose; no bleeding was observed in other mice, including those receiving a 10% bolus injection with continuous infusion of the remaining 90%, which was most effective. A previous report demonstrated that an increase in vascular permeability induced by vascular endothelial growth factor was inhibited by monoclonal antibodies that block integrin α_v_β_5_ [37]. Saxatilin showed high affinity for integrin α_v_β_5_ in our study; thus, saxatilin might be beneficial as an α_v_β_5_ antagonist for preventing bleeding complications induced by vascular leakage. Additionally, α_v_β_5_ inhibition following administration of saxatilin might be advantageous for stroke outcomes. In β_5_-deficient mice, infarction volume and blood-brain barrier breakdown is dramatically reduced after permanent middle cerebral artery occlusion [37]. 

We also determined the effect of saxatilin on aged thrombus. Thrombolytic effects of saxatilin decreased as thrombus age increased. Decreased thrombolytic effects in aged thrombi were also seen in rt-PA. While effects of thrombolytic drugs on aged thrombi have not well known, our findings suggest that effects of drugs acting on platelets as well as fibrinogen may be decreased as thrombus age increases and that reducing time from onset to treatment is important to enhance effects of the thrombolytic drugs.

Our methods had distinct features compared to other experimental models evaluating thrombolytic effects. First, to produce arterial thrombosis, we used a smaller filter paper saturated with a higher concentration (50%) of FeCl_3_ than previous studies (2.5–65%) [23,24,38,39]. This was because we needed to monitor blood flow for a longer period to examine the thrombolytic effects of drugs. In addition, the smaller thrombi produced from smaller filter papers were better for monitoring blood flow, examining pathology after drug treatment, and determining drug-induced thrombolytic effects. When we used low concentrations of FeCl_3_ such as 10-40%, blood flow restored spontaneously during 150 minutes of monitoring period after initially complete occlusion. Low concentrations of FeCl_3_ are usually used to evaluate thrombosis mechanisms or the effects of antithrombotic drugs, which do not require the long-term monitoring needed to evaluate thrombolytic effects. Scanning and transmission electron microscopy showed that 50% FeCl_3_ causes a loss of undulation and severe damage to the endothelium and smooth muscle but did not damage internal elastic lamina. These findings were consistent with previous observations using a lower dose of FeCl_3_ (7.5-20%) [38]. 

Second, the assessment methods we used were different from previous studies in which recanalization is typically assessed to determine thrombolytic effects. Our study determined and calculated the degree of flow restoration on a minute-by-minute basis using ultrasound blood flow measurements. Histological examination showed that blood flow measurements were a good representation of the degree of recanalization and thrombus resolution. The assessment method we used provided quantitative data on degree of recanalization, time to effective recanalization, and real-time occurrence of reocclusion. Our method also enabled group comparisons as well as quantitative data on each animal. We found a dose-dependent thrombolytic effect for rt-PA. The effective dose of rt-PA in rodents is about 10-fold higher than for humans because of different PA systems [40-42]. Therefore, 9 mg/kg, which gave optimal thrombolytic effects in mice in our study, is comparable to 0.9 mg/kg in humans. Thus, this assessment method might be a reliable tool for evaluating the *in vivo* efficacy of new thrombolytic drugs. 

Third, thrombolytic effects were determined using platelet aggregometry. Platelet aggregometry has been used to test inhibition of thrombus formation and is typically used to assess platelet anti-aggregating drugs. However, we used platelet aggregometry to assess platelet disaggregation after administering a thrombolytic drug to preformed thrombi. We found that platelet aggregometry could also be used to assess *in vitro* thrombolytic effects. 

In conclusion, disaggregation of platelets from fibrin is a potential approach to dissolving thrombi [11,12]. Several specific GP IIb/IIIa receptor inhibitors have been developed and are currently clinically available. However, saxatilin, which originates from natural sources and inhibits multiple integrins that act on platelets, could be a candidate for a new thrombolytic drug with improved potency. 
